# The expression of PD-1 ligands in the immune microenvironment was altered in TTF-1-negative lung adenocarcinoma

**DOI:** 10.1007/s13577-025-01275-y

**Published:** 2025-08-18

**Authors:** Hiroyuki Yamada, Hiromu Yano, Eri Matsubara, Shukang Zhao, Yusuke Shinchi, Cheng Pan, Takamasa Koga, Kosuke Fujino, Yukio Fujiwara, Koei Ikeda, Yoshihiro Komohara, Makoto Suzuki

**Affiliations:** 1 Department of Cell Pathology, Graduate School of Medical Sciences, Kumamoto University, 1-1-1, Honjo, Chuo-Ku, Kumamoto, 860-8556 Japan; 2 Department of Thoracic Surgery and Breast Surgery, Faculty of Life Sciences, Kumamoto University, 1-1-1, Honjo, Chuo-Ku, Kumamoto, 860-8556 Japan; 3 Center for Metabolic Regulation of Healthy Aging, Kumamoto University, 1-1-1, Honjo, Chuo-Ku, Kumamoto, 860-8556 Japan

**Keywords:** Lung adenocarcinoma, TTF-1, NKX2-1, Macrophage, PD-L1

## Abstract

Thyroid transcription factor-1 (TTF-1) is a lineage-specific marker for lung adenocarcinoma (LUAD), whereas the relatively minor subset of TTF-1-negative LUADs shows a poor prognosis and a limited response to therapy. However, its relationship with the tumor immune microenvironment remains poorly defined. How TTF-1 expression affects the immune context in LUAD was investigated, focusing on tumor-associated macrophages (TAMs) and T-cell infiltration. Immunohistochemical (IHC) analysis of 226 LUAD specimens showed that TTF-1-negative tumors were associated with epidermal growth factor receptor wild-type status, advanced stage, and worse progression-free and cancer-specific survivals. Notably, PD-L1 (programmed death-ligand 1) and PD-L2 expression in TAMs, but not in cancer cells, was significantly reduced in TTF-1-negative tumors. Public single-cell RNA sequencing data confirmed downregulation of *CD274* (PD-L1) in TAMs from tumors with low expression of TTF-1-related genes. In contrast, *PDCD1LG2* (PD-L2) expression showed less consistent patterns. On IHC analysis, infiltration of CD8^+^ and CD4^+^ T cells was modestly lower in TTF-1-negative tumors, accompanied by decreased HLA class I and II expressions. Transcriptomic analysis of The Cancer Genome Atlas LUAD cohort further showed lower interferon gamma (IFN-γ) signaling and decreased T cell-inflamed gene signatures in the low TTF-1-negative tumors. These findings suggest that TTF-1-negative LUAD exhibits more immune-suppressive features, with a relatively reduced antitumor immune response characterized by decreased T-cell infiltration and INF-γ signaling, which are related to PD-L1 and PD-L2 expressions in TAMs.

**Supplementary Information:**

The online version contains supplementary material available at 10.1007/s13577-025-01275-y.

## Introduction

Lung cancer remains the leading cause of cancer-related deaths worldwide, with most cases still diagnosed at advanced stages despite improvements in early detection using computed tomography [1, 2]. In recent years, immune checkpoint inhibitors (ICIs), particularly those targeting programmed cell death-1 (PD-1) and its ligand PD-L1 (programmed death-ligand 1), have become a standard treatment and have shown significant efficacy in selected lung cancer patients [3, 4].

The PD-L1/PD-1 interaction occurring between tumor cells and CD8^+^ T cells leads to T cell exhaustion and immune tolerance [5]. PD-L1 expression in lung adenocarcinoma (LUAD) cells serves as a key biomarker for predicting ICI efficacy [6, 7]. Tumor-associated macrophages (TAMs), which are abundant in the tumor immune microenvironment (TIME), also express PD-L1 and PD-L2, both ligands of PD-1, and contribute to immunosuppression [8–10]. Our previous studies further showed that PD-L1/PD-L2 expression in TAMs is associated with clinical prognosis in LUAD [11, 12].

Thyroid transcription factor-1 (TTF-1, also known as NKX2-1) is a lineage-specific transcription factor expressed in distal lung epithelial cells, including type II alveolar and Clara cells [13]. It plays essential roles in lung development and surfactant gene regulation [14, 15], and it is also expressed in the majority of LUADs, serving as a diagnostic marker [16, 17]. Beyond its critical functions in normal lung, TTF-1 exerts both oncogenic and tumor-suppressive roles in LUAD [18]. These include promoting tumor growth via the PI3K-AKT pathway through ROR1, especially in epidermal growth factor receptor (EGFR)-mutant LUAD [19], and suppressing tumor progression by upregulating adhesion-related genes (e.g., *MYBPH*, *OCLN*, *CLDN1*, *CLDN18*) and inhibiting the epithelial-mesenchymal transition and KRAS-driven mucinous transformation [20–24]. Clinically, TTF-1-negative LUAD, representing approximately 20–30% of cases, is consistently associated with more aggressive behavior, including poorer prognosis and reduced sensitivity to pemetrexed and ICIs [25–31].

Although these findings highlight the dual roles of TTF-1 in LUAD progression, its association with the TIME remains poorly understood. In particular, little is known about how TTF-1 status influences TAMs and CD8^+^ T cells. In this study, PD-L1 and PD-L2 expressions in TAMs were found to be decreased in TTF-1-negative LUAD.

## Materials and methods

### Samples

Formalin-fixed, paraffin-embedded tumor tissues from 226 LUAD patients diagnosed between 2010 and 2013 at Kumamoto University Hospital were analyzed. Tissue microarrays prepared previously by our group were used for immunohistochemical (IHC) analysis [32].

### IHC analysis

IHC was performed on paraffin-embedded sections using the following primary antibodies: anti-TTF-1 (clone 8G7G3/1; Dako, Agilent Technologies, Santa Clara, CA, USA), anti-CD3 (clone SP7; Nichirei, Tokyo, Japan), anti-CD8 (clone C8/144B; Nichirei), anti-FOXP3 (clone 236A/E7; Abcam, Cambridge, UK), anti-HLA-A/B/C (clone EMR8-5; Medical & Biological Laboratories, Tokyo, Japan), and anti-HLA-DR (clone TAL1B5; Dako, Agilent Technologies). Sections were incubated with horseradish peroxidase-labeled secondary antibodies (anti-mouse: #424131; anti-rabbit: #R24141; Histofine, Nichirei) and visualized with a 3,3’-diaminobenzidine (DAB) substrate kit (#425011; Nichirei). For double staining of CD3 and CD8, sections were first stained for CD3 using DAB, then subjected to heat treatment in 1 mmol/L EDTA buffer (pH 8.0), followed by CD8 staining and visualization using HistoGreen substrate (#AYS-E109; Linaris, Heidelberg, Germany). CD4 is known to be expressed not only on lymphocytes, but also on macrophages, which can complicate the interpretation of its staining in tumor tissue. Therefore, CD3 and CD8 were used to identify T lymphocytes, and CD3^+^/CD8^−^ cells were interpreted as CD4^+^ T cells. Two investigators (Y.K. and H.Y.), blinded to clinical information, independently evaluated all IHC-stained sections. Data on PD-L1 and PD-L2 expressions in cancer cells and TAMs were previously reported [11, 12]. In this study, the percentage of PD-L1^+^ or PD-L2^+^ cells among PU.1^+^ macrophages was defined as the macrophage proportion score (MPS); scores < 50% were categorized as low expression, and ≥ 50% as high expression. For selected cases, multichannel pseudocolored images were generated using HALO (version 4.0.5107.318; Indica Labs, Albuquerque, NM, USA) and NanoZoomer S20 (Hamamatsu Photonics, Hamamatsu, Japan), based on color deconvolution. For HLA expression, staining intensity of HLA-A/B/C and HLA-DR in tumor cells was classified as strong, moderate, weak, and complete loss. The expression in alveolar epithelial cells and macrophages was used as the internal reference. HLA class I was considered preserved when staining was uniformly strong; any area with reduced intensity was classified as reduced. For HLA-DR, cases with complete retention were defined as preserved, and any area of complete loss was considered reduced [33].

### Single-cell RNA-seq analysis

Single-cell RNA-sequence (scRNA-seq) data from two LUAD datasets (GSE162498 and GSE131907, total 20 patients) were obtained from the Gene Expression Omnibus [34, 35]. Data were processed using Seurat (version 5.1.0) and Harmony in R (version 4.4.0; R Foundation for Statistical Computing, Vienna, Austria) to account for batch effects [36, 37]. Low-quality cells (≤ 200 or ≥ 5000 genes or ≥ 15% mitochondrial content) were excluded. Each sample was converted to a Seurat object and merged using the merge function, followed by layer integration (JoinLayers), normalization (NormalizeData), and variable gene selection (FindVariableFeatures). Gene expression was scaled using ScaleData, and batch correction was performed with RunHarmony based on orig.ident. Dimensionality reduction and clustering were done using principal component analysis (PCA), FindNeighbors, and FindClusters at a resolution of 1.0, yielding 28 clusters. Tumor cells were identified based on inferred copy number variation (CNV) following Kim et al. [35]; that is, epithelial cells were evaluated using two scores: the mean squared (MS) score (reflecting chromosomal variability) and the correlation (COR) score (similarity to tumor reference). Cells with MS > 0.02 or COR > 0.2 were classified as tumor cells. Immune cells were then extracted and re-clustered using 9 PCs and a resolution of 0.8. Within this subset, myeloid cells were further isolated and re-clustered using 14 PCs and a resolution of 1.0. Cells expressing canonical macrophage markers and lacking features of low-quality or uncertain identity were annotated as macrophages. The AddModuleScore function was used to calculate per-cell expression scores for predefined gene sets. To score TTF-1 target genes, genes reported to be upregulated by TTF-1 [18], including surfactant proteins (*SFTPA1*, *SFTPA2*, *SFTPB*, *SFTPC*), secretoglobins (*SCGB1A1*, *SCGB3A2*), *ABCA3*, and *PDPN*, were used.

### Bulk RNA-seq analysis of TCGA-LUAD

A total of 516 LUAD cases were analyzed using R (version 4.4.0), based on transcriptome data obtained from The Cancer Genome Atlas (TCGA) official data portal (https://portal.gdc.cancer.gov/). When multiple specimens were available for a case, the sample with the lowest alphabetical vial label (i.e., vial A) and smallest numerical portion number was selected. Single-sample gene set enrichment analysis (ssGSEA) was performed using the GSVA package, with parameters set via the ssgseaParam function. Expression of TTF-1 target genes (as defined in the scRNA-seq analysis) was quantified by ssGSEA, and cases were divided into high and low groups by median values. Immune cell-type abundance was estimated using the xCell algorithm via the immunodeconv package [38]. The 18-gene T cell-inflamed gene expression profile (GEP), a validated predictor of response to PD-1 blockade, was also evaluated [39]. The HLA class I signature included *HLA-A*, *HLA-B*, *HLA-C*, and *HLA-E*, whereas class II comprised *HLA-DRA*, *HLA-DRB1*, and *CIITA*. An interferon gamma (IFN-γ) response signature corresponding to the HALLMARK_INTERFERON_GAMMA_RESPONSE gene set from MsigDB was also assessed [40].

### Statistical analysis

Statistical analyses were conducted using R (version 4.4.0). Fisher’s exact test was used for categorical variable comparisons between two groups, and the Mann–Whitney *U* test was applied to continuous variables not assumed to follow a normal distribution. Overall survival was analyzed using the Kaplan–Meier method with log-rank tests. For comparisons of T-cell densities, nonparametric effect sizes (*r*) were calculated from Mann–Whitney *U* statistics. Post hoc statistical power was estimated using the pwr package in R. A *p*-value of < 0.05 was considered statistically significant [41].

## Results

### TTF-1-negative cases show a worse clinical course in LUAD

**Fig. 1:**
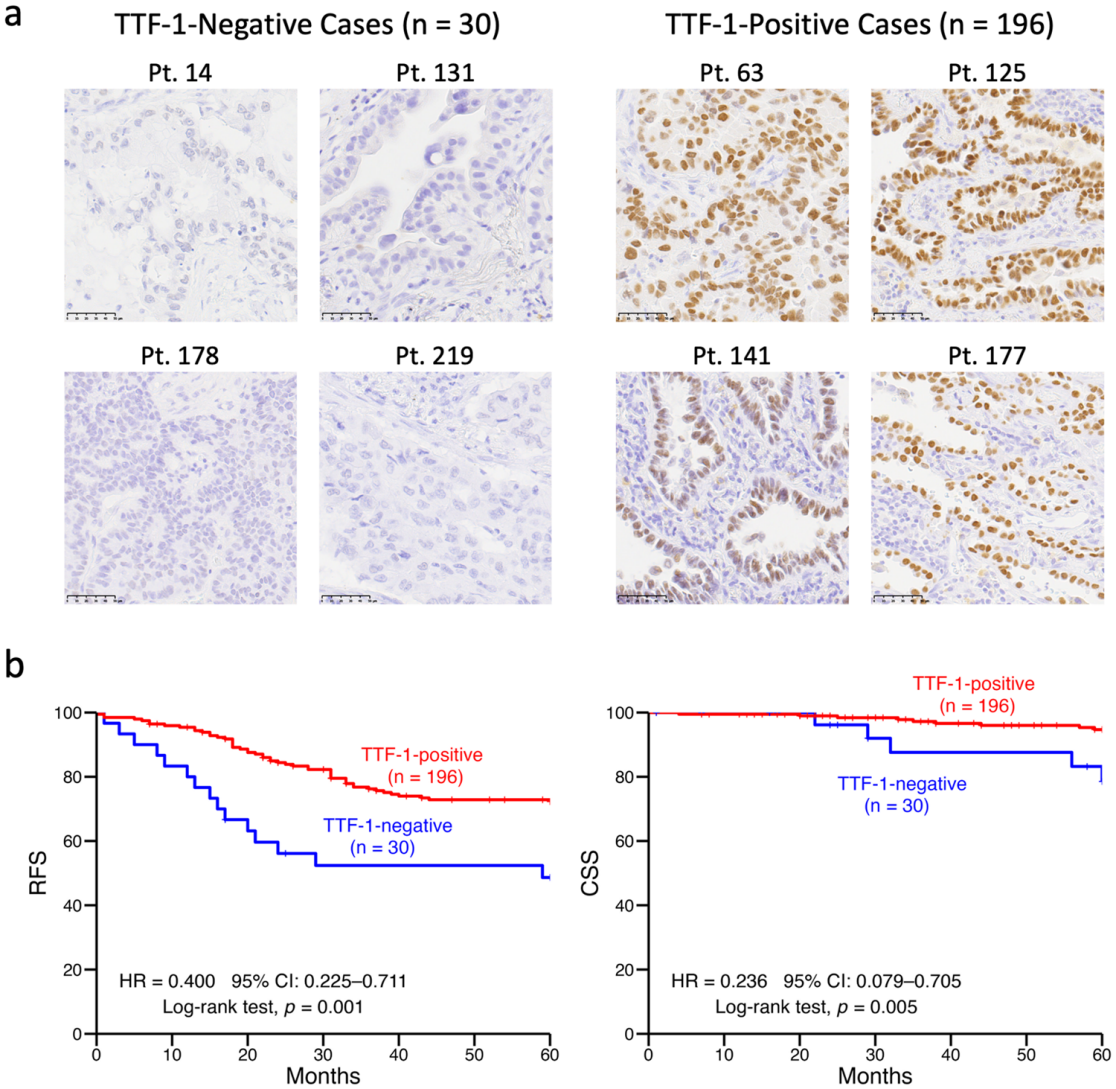
Thyroid transcription factor-1 (TTF-1) expression and its clinical significance. **a** Representative immunohistochemical (IHC) images of TTF-1 expression from 226 lung adenocarcinoma (LUAD) cases (scale bars: 50 µm). **b** Kaplan–Meier analysis of recurrence-free survival (RFS) and cancer-specific survival (CSS) by TTF-1 status. Log-rank tests were performed, and hazard ratios (HRs) with their 95% confidence intervals (CIs) were calculated using Cox regression analyses

On IHC analysis, 196 cases (86.7%) were TTF-1 positive, and 30 cases (13.3%) were negative. Representative images of positive and negative staining are shown in Fig. 1a. TTF-1 negativity was significantly associated with EGFR wild-type status, male sex, smoking, and advanced disease stage (Table 1). Of EGFR-mutant tumors, nearly all (98.1%) were TTF-1 positive. Kaplan–Meier analysis showed that both recurrence-free survival (RFS) and cancer-specific survival (CSS) were significantly worse in TTF-1-negative cases (Fig. 1b). Given the clear difference in RFS, univariate and multivariate Cox regression analyses were performed (Supplementary Table 1). On univariate analysis, factors associated with TTF-1-negativity, including male sex, smoking, and advanced stage, were significantly associated with shorter RFS. On multivariate analysis including these variables along with EGFR mutation status, only advanced stage remained an independent prognostic factor, whereas TTF-1 expression was not. However, in a separate multivariate analysis excluding pathological stage, TTF-1 negativity remained the only independent predictor of poor RFS (adjusted HR 0.42, 95% CI: 0.23–0.79, *p* = 0.007).

**Table 1:** TTF-1 expression and clinicopathological factors

	TTF-1 expression		
	Negative	Positive	*p*-value
*EGFR gene status*			
Wild type	26 (92.9)	82 (43.9)	< 0.001
Mutant	2 (7.1)	105 (56.1)	
*Age*			
< 65	5 (16.7)	61 (31.1)	0.13
≥ 65	25 (83.3)	135 (68.9)	
*Gender*			
Male	22 (73.3)	91 (46.4)	0.010
Female	8 (26.7)	105 (53.6)	
*Smoking*			
BI < 600	9 (30.0)	131 (66.8)	< 0.001
BI ≥ 600	21 (70.0)	65 (33.2)	
*Pathological stage*			
0–I	16 (53.3)	158 (80.6)	0.002
II–VI	14 (46.7)	38 (19.4)	
*Grade*			
1,2	23 (76.7)	190 (96.9)	< 0.001
3	7 (23.3)	6 (3.1)	
*PD-L1 expression on cancer cells*			
Low: < 50%	26 (86.7)	181 (92.3)	0.29
High: ≥ 50%	4 (13.3)	15 (7.7)	
*PD-L1 expression on TAMs*			
Low: < 50%	22 (73.3)	88 (44.9)	0.005
High: ≥ 50%	8 (26.7)	108 (55.1)	
*PD-L2 expression on cancer cells*			
Low: < 50%	23 (76.7)	124 (63.3)	0.22
High: ≥ 50%	7 (23.3)	72 (36.7)	
*PD-L2 expression on TAMs*			
Low: < 50%	22 (73.3)	75 (38.3)	< 0.001
High: ≥ 50%	8 (26.7)	121 (61.7)	

Fisher’s exact test was performed. Underline indicates statistically significant

*TTF-1*, thyroid transcription factor-1; *EGFR*, epidermal growth factor; *BI*, Brinkman index; *PD-L1/L2*, programmed cell death-1 ligand 1 and 2; TAMs, tumor-associated macrophages

### TTF-1-negative cases show lower expression of PD-L1 and PD-L2 in TAMs

**Fig. 2:**
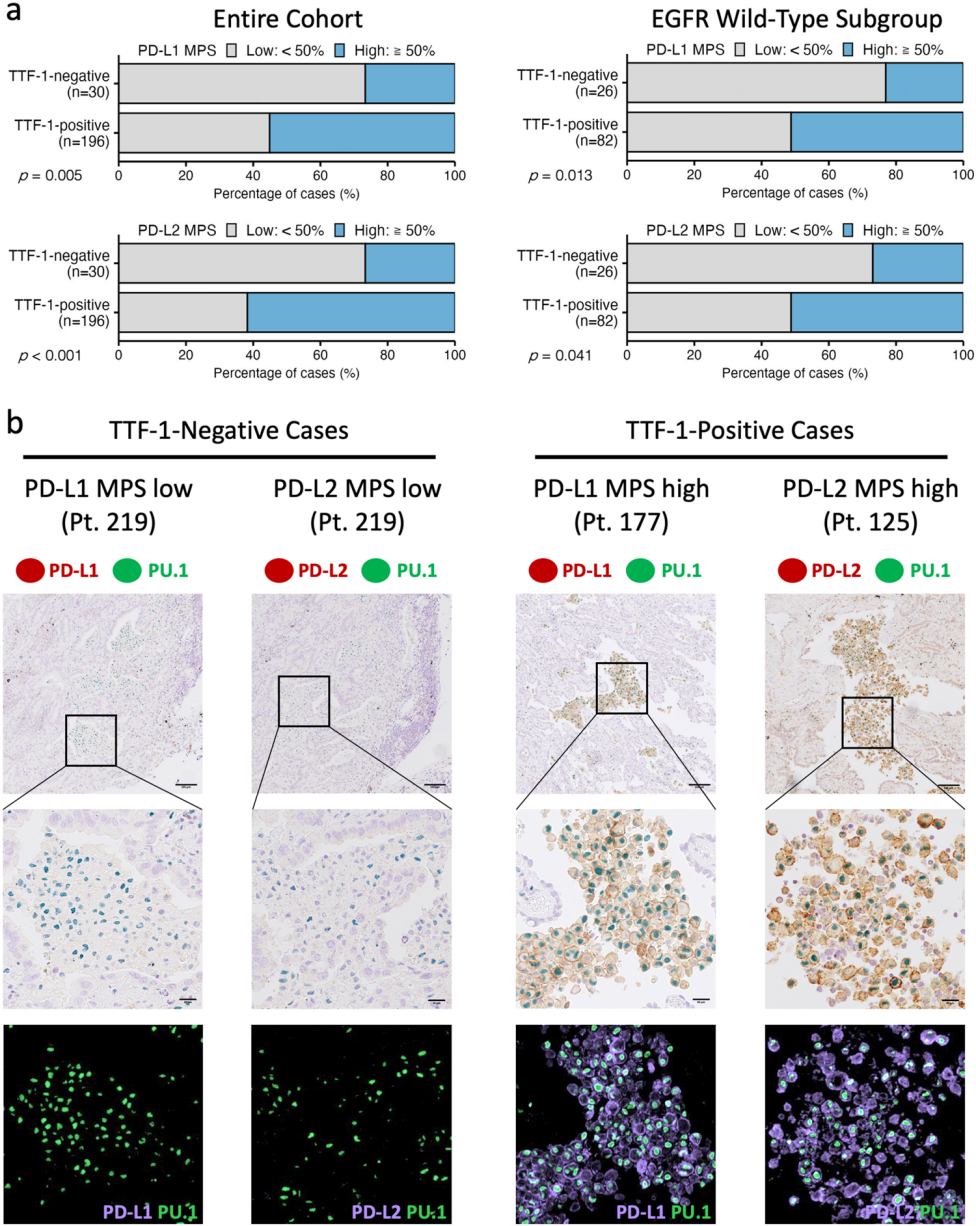
TTF-1 status and PD-L1/L2 expression in TAMs. **a** Proportions of cases with high PD-L1 or PD-L2 MPS (≥ 50%) are shown for the entire cohort and for the EGFR wild-type subgroup. Group comparisons were performed using Fisher’s exact test. **b** Representative images of TAMs in TTF-1-negative and TTF-1-positive cases. Top row: chromogenic double IHC (scale bars: 100 µm); middle row: magnified chromogenic images (scale bars: 20 µm); bottom row: pseudocolored images. Pseudocoloring was performed using the color deconvolution function in HALO software: PU.1 (green), PD-L1/PD-L2 (blue-magenta). *PD-L1*, programmed cell death-1 ligand 1; *PD-L2*, programmed cell death-1 ligand 2; *TAMs*, tumor-associated macrophages; *MPS*, macrophage proportion score; *EGFR*, epidermal growth factor receptor

The relationship between TTF-1 status and PD-L1/PD-L2 expression was next examined using previously reported IHC data from the same tissue microarray sections (see Sect."IHC analysis"). No significant correlation was observed between TTF-1 status and PD-L1/PD-L2 expression in cancer cells (Table 1). In contrast, a significant association was found in TAMs, quantified as the MPS score (Table 1 and Fig. 2a). The proportion of cases with high PD-L1 MPS was significantly lower in TTF-1-negative tumors than in TTF-1-positive tumors. This association remained significant within the EGFR wild-type subgroup. Similarly, high PD-L2 MPS was less frequent in TTF-1-negative cases overall and within the EGFR wild-type subgroup. Representative images of co-immunostaining for PD-L1 or PD-L2 with PU.1 are shown in Fig. 2b. Pseudocolored images further illustrate the expressions of these proteins in macrophages. Multivariate logistic regression analysis including variables that were significant on univariate analysis demonstrated that TTF-1 positivity was independently associated with high PD-L1 MPS (Supplementary Table 2). In contrast, high PD-L2 MPS was independently associated only with early-stage disease, and not with TTF-1 status (Supplementary Table 3). These findings suggest that PD-L2 expression in TAMs decreases with tumor progression, and that its association with TTF-1 may be indirect or stage-dependent.

### scRNA-seq data indicates downregulation of PD-L1 in TAMs from TTF-1-negative cases

**Fig. 3:**
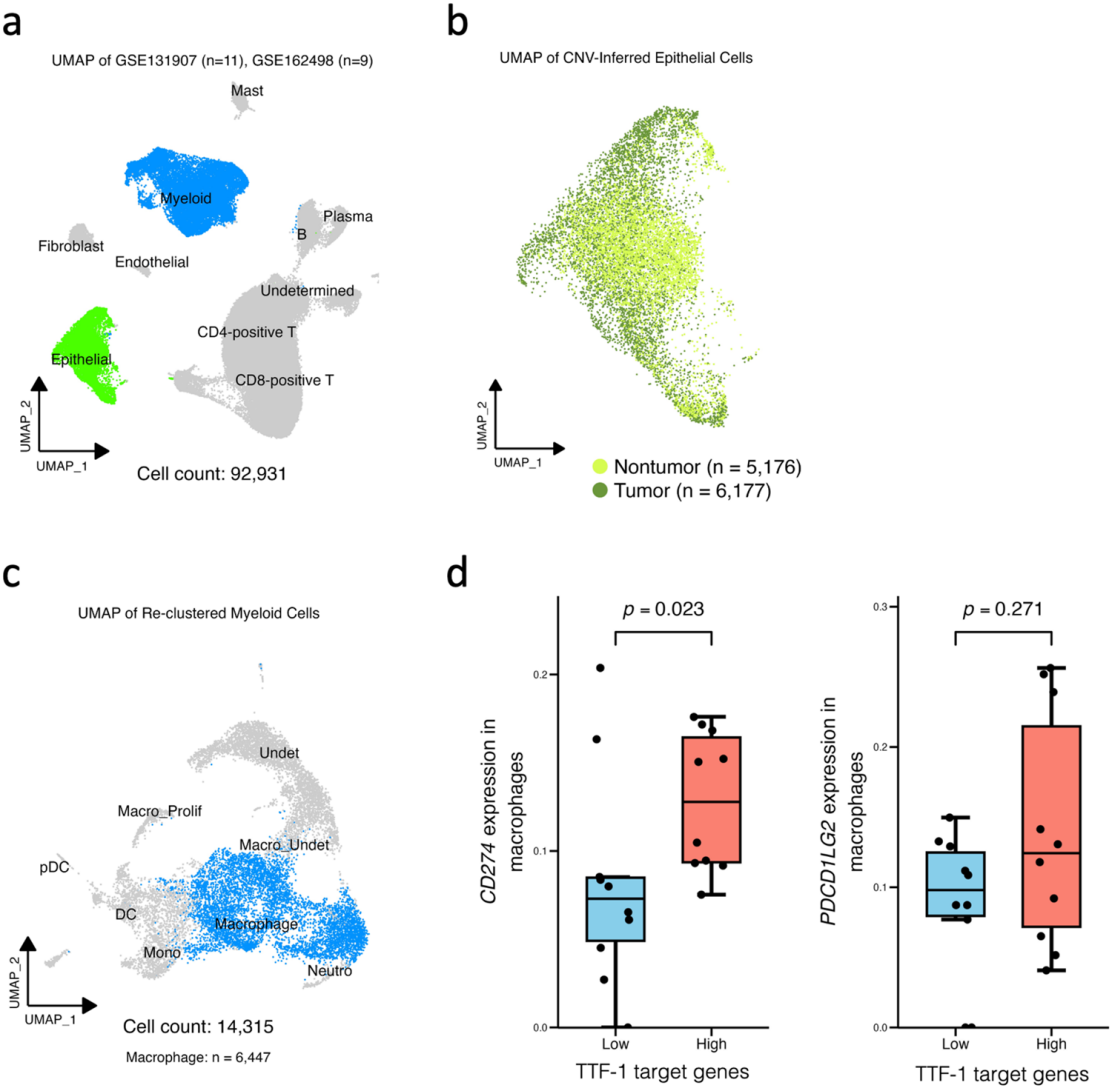
Single-cell RNA sequencing analysis of public LUAD data. **a** Data from 20 LUAD patients were analyzed and visualized by UMAP following clustering. **b** Tumor epithelial cells were identified by inferred CNV analysis. For clarity, cells above the 99.75th percentile on the x-axis were omitted from the plot but included in downstream analyses. **c** Myeloid cells were extracted and re-clustered from immune cells. Clusters expressing canonical macrophage markers with undetermined identities were classified as macrophages. **d** For each patient, tumor cells were evaluated for expression of a predefined TTF-1 target gene set. Patients were stratified into high and low groups based on the median values. Mean expression levels of *CD274* (encoding PD-L1) and *PDCD1LG2* (encoding PD-L2) in macrophages were compared between the two groups using the Mann–Whitney *U* test. *UMAP*, Uniform Manifold Approximation and Projection; *CNV*, copy number variations; UMI, unique molecular identifier

To validate the IHC findings regarding PD-L1/PD-L2 expression in TAMs, publicly available scRNA-seq data from 20 LUAD patients were reanalyzed (Fig. 3a). Epithelial cells were subclassified into tumor and non-tumor cells based on inferred CNV (Fig. 3b). Myeloid cells were extracted and reclustered, and those expressing canonical macrophage markers were defined as macrophages (Fig. 3c). Because *NKX2-1* expression was generally low and subject to technical variability at the single-cell level, the average expression score of eight known TTF-1 target genes was instead quantified. The low TTF-1 target gene group was defined as the TTF-1-negative cases. Patients were stratified into low and high groups based on the median score. The average expression of *CD274* (encoding PD-L1) in macrophages was significantly lower in the low TTF-1 target gene group than in the high group (Fig. 3d). In contrast, *PDCD1LG2* (encoding PD-L2) expression did not differ significantly between the groups. These results are consistent with the IHC findings for PD-L1, but not for PD-L2, and they support the association between TTF-1 activity and PD-L1 expression in TAMs. In contrast, when stratifying by *NKX2-1* expression itself, no significant differences in *CD274* and *PDCD1LG2* were observed (Supplementary Fig. 1).

### Infiltration of T-cell subsets is modestly reduced in TTF-1-negative cases

**Fig. 4:**
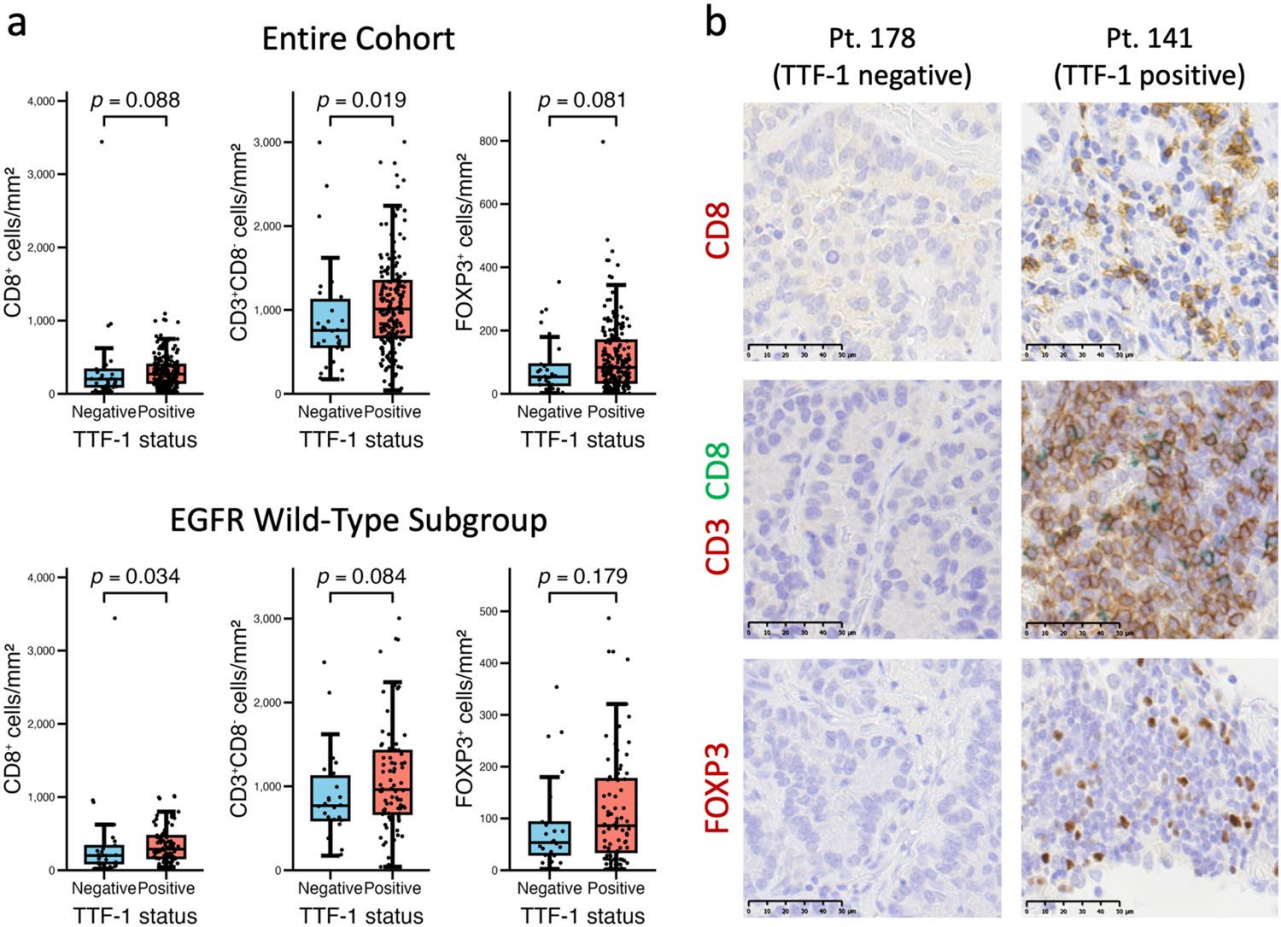
T-cell subset densities according to TTF-1 status. **a** Densities of CD8^+^ T cells, CD3^+^CD8^−^ T cells (interpreted as CD4^+^ T cells), and FOXP3.^+^ T cells are compared between TTF-1-negative and TTF-1-positive tumors, in both the entire cohort and the EGFR wild-type subgroup. Comparisons were performed using the Mann–Whitney *U* test. **b** Representative IHC images of CD8, CD3/CD8, and FOXP3 staining from a TTF-1-negative case and a TTF-1-positive case (scale bars: 50 µm)

T-cell infiltration was next assessed by evaluating subset-specific densities using IHC. In the entire cohort, CD8^+^ T-cell density showed a nonsignificant trend toward reduction in TTF-1-negative tumors. However, within the EGFR wild-type subgroup, CD8^+^ T-cell density was significantly lower in TTF-1-negative cases than in TTF-1-positive cases (Fig. 4a, left panels). The density of CD3^+^CD8^−^ T cells, interpreted as CD4^+^ T cells in this study, was significantly lower in TTF-1-negative tumors in the overall cohort. A similar, though nonsignificant, trend was observed in the EGFR wild-type subgroup (Fig. 4a, center panels). FOXP3^+^ T-cell density did not differ significantly between TTF-1 status groups, although TTF-1-negative tumors tended to show lower counts (Fig. 4a, right panels). However, these differences were generally modest. For CD8^+^ T cells in the overall cohort, the effect size was small (*r* = 0.11), and the statistical power was low (0.015). In the EGFR wild-type subgroup, although the effect size was slightly higher (*r* = 0.21), the statistical power remained limited (0.050). Therefore, these differences were regarded as exploratory and interpreted in the context of other experimental results. Representative IHC images of CD8, CD3/CD8, and FOXP3 staining in a TTF-1-negative case and a TTF-1-positive case are shown in Fig. 4b.

### HLA class I and II expression in tumor cells is reduced in TTF-1-negative cases

**Fig. 5:**
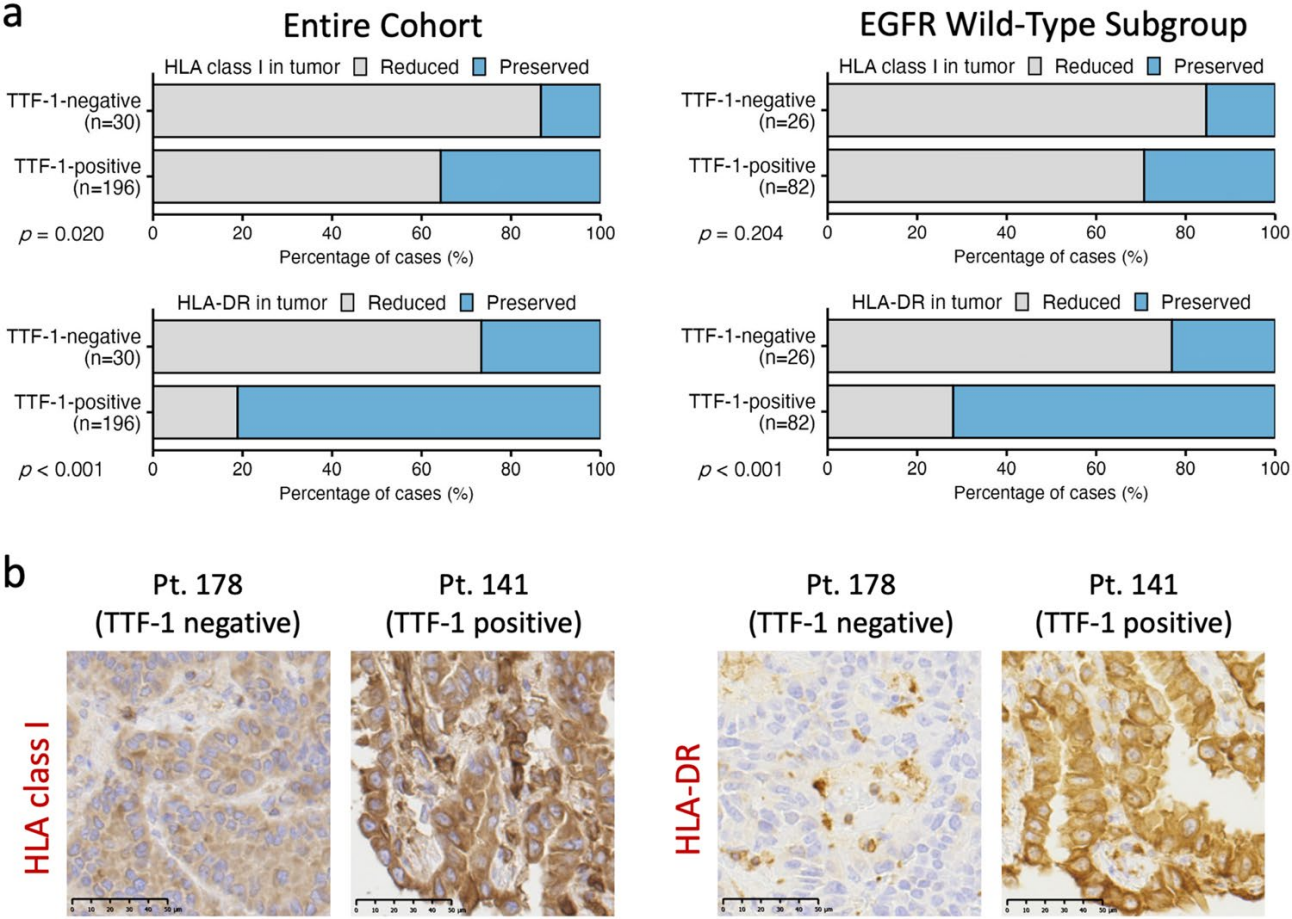
HLA class I and II expressions in tumor cells according to TTF-1 status. **a** Frequencies of preserved and reduced expressions of HLA class I (HLA-A/B/C, upper) and HLA-DR (lower) in tumor cells are shown for the entire cohort (left) and the EGFR-wild-type subgroup (right). Classification into preserved and reduced was based on staining intensity and distribution pattern, as described in Materials and Methods. Differences between TTF-1-positive and TTF-1-negative groups were evaluated using Fisher’s exact test. **b** Representative IHC images of HLA class I and HLA-DR in tumor cells from a TTF-1-negative case and a TTF-1-positive case. Stromal cells such as immune cells are positive for HLA class I and HLA-DR, and these positive signals were evaluated as internal positive controls (scale bars: 50 µm)

Since HLA-class I and II expressions in cancer cells are critical for anti-tumor immune responses, protein expressions of HLA class I (HLA-A/B/C) and class II (HLA-DR) were evaluated in tumor cells using IHC. HLA class I expression in tumor cells was decreased, and this was significantly more frequent in TTF-1-negative cases than in TTF-1-positive cases. A similar trend was observed in the EGFR wild-type subgroup, although it was not significant (Fig. 5a, upper panels). HLA-DR expression showed a more pronounced pattern. TTF-1-positive tumors retained strong expression, whereas TTF-1-negative tumors exhibited marked and significant loss of HLA-DR in both the entire cohort and the EGFR wild-type subgroup (Fig. 5a, lower panels). Representative IHC images from TTF-1-negative and TTF-1-positive tumors are shown in Fig. 5b.

### *Bulk RNA-seq data suggests lower IFN-*γ* signaling in TTF-1-negative tumors*

**Fig. 6:**
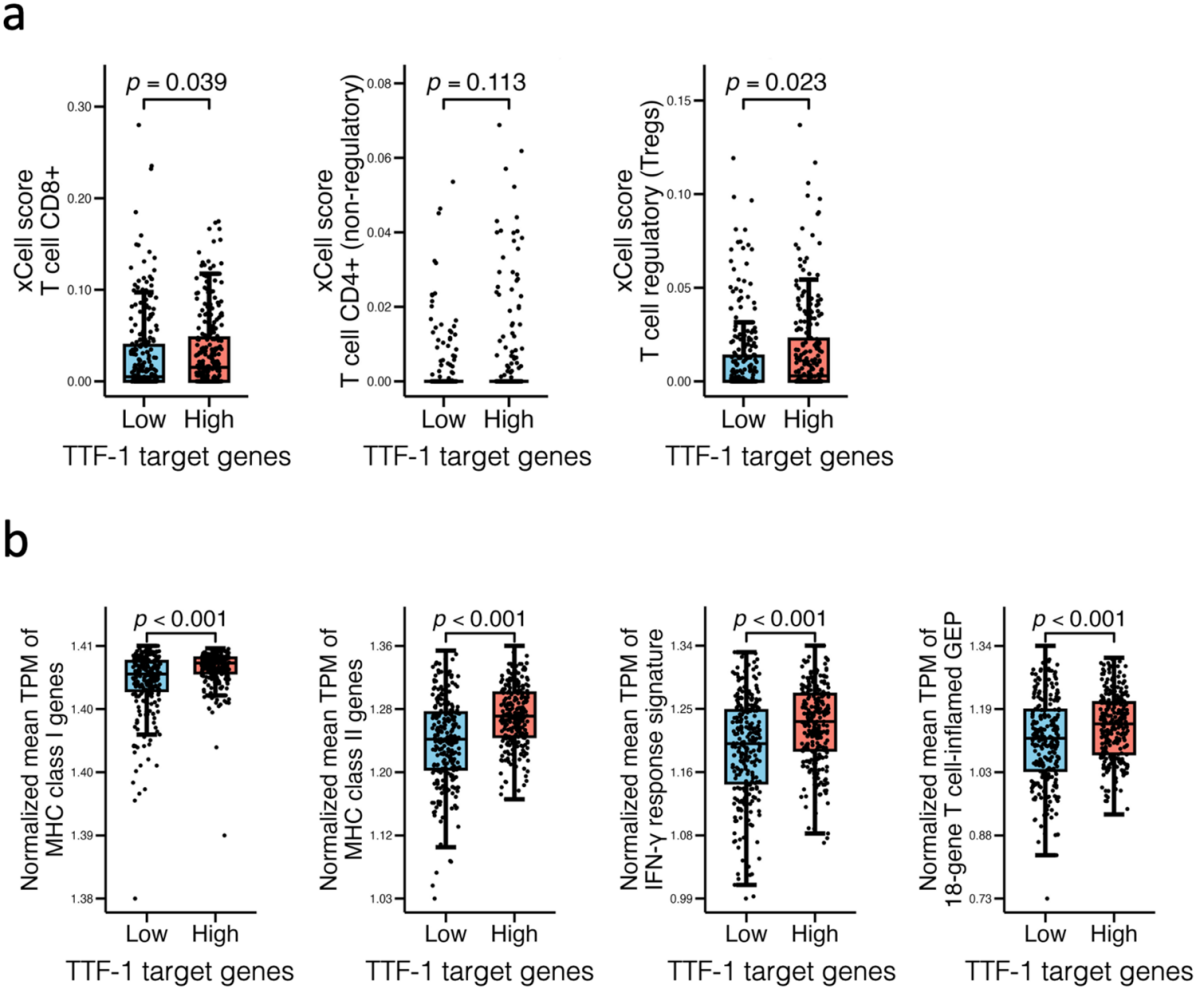
Immune composition and gene expression in TCGA-LUAD by TTF-1 target gene score. **a** xCell scores for CD8^+^ T cells, non-regulatory CD4^+^ T cells, and regulatory T cells (Tregs) are shown for comparisons. **b** Normalized expression levels of HLA class I genes (*HLA-A*, *-B*, *-C*, *-E*), class II genes (*HLA-DRA*, -*DRB1*, *CIITA*), the IFN-γ response signature, and the 18-gene T cell-inflamed gene expression profile (GEP) are shown. All gene signatures were quantified by ssGSEA, and cases were divided at the median value of the TTF-1 target gene score. Comparisons were performed using the Mann–Whitney *U* test. *TCGA*, The Cancer Genome Atlas; *ssGSEA*, single-sample gene set enrichment analysis

To further characterize the TIME, bulk RNA-seq data from 516 LUAD cases in the TCGA-LUAD dataset were analyzed. Cases were stratified into two groups based on the median expression score of TTF-1 target genes, as in the scRNA-seq analysis. Immune cell composition was inferred using xCell. The CD8^+^ T-cell score was significantly lower in the TTF-1-negative group, consistent with the IHC findings (Fig. 6a). The non-regulatory CD4^+^ T-cell score was generally low and showed no significant difference. In contrast, Treg scores were significantly higher in the TTF-1-positive group. Expressions of HLA class I and II genes were also significantly lower in the TTF-1-negative group, in agreement with the IHC results (Fig. 6b). The IFN-γ response signature and the 18-gene T cell-inflamed GEP were both significantly reduced in the TTF-1-negative group. These results support the notion that antitumor immune activity is attenuated in TTF-1-negative tumors.

## Discussion

This study demonstrates that TTF-1-negative LUAD exhibits features of immune evasion and reduced immunogenicity. Consistent with previous reports, TTF-1-negative tumors were associated with a significantly worse prognosis, largely mediated by the advanced stage. Although TTF-1 or its gene *NKX2-1* functions both as a lineage oncogene and a tumor suppressor, its role in the TIME has been less studied. It was found that TTF-1-negative LUAD showed decreased CD8^+^ and CD4^+^ T-cell infiltration and impaired antigen presentation, particularly loss of HLA-DR. Supporting this, TCGA data showed that the TTF-1-negative group had decreased HLA gene expression and decreased IFN-γ and T cell-inflamed signatures, pointing to a less inflamed TIME phenotype.

A key observation was reduced PD-L1 and PD-L2 expressions in TAMs from TTF-1-negative tumors, whereas no such difference was seen in cancer cells. Although a functional link between TTF-1 and EGFR mutation is suggested [18], including by the present data, multivariate and EGFR wild-type subgroup analyses indicate that the association is at least partially independent of EGFR. A similar association between lower *CD274* expression in TAMs and reduced TTF-1 activity was also observed on scRNA-seq analysis. Since PD-L1/PD-L2 expression in TAMs is mainly induced by cytokines such as IFN-γ secreted from activated CD8^+^ T cells and CD4^+^ helper type 1 cells [42, 43], its downregulation may be predominantly driven by reduced IFN-γ signaling, as suggested in TTF-1-negative tumors in the present analysis. In contrast, PD-L1 expression in tumor cells is often driven by intrinsic genetic alterations such as amplification [44, 45], potentially explaining its lack of association with TTF-1.

Clinically, PD-L1 expression in TAMs has been linked to ICI efficacy in melanoma and ovarian cancer [10], but it is not routinely assessed. TTF-1 immunostaining, being widely available, may serve as a surrogate predictor of ICI response and a more inflamed TIME, especially when tumor cell PD-L1 is low. Nakahama et al. reported poor ICI monotherapy responses in TTF-1-negative LUAD [27]. Several studies have shown that combining ICIs with chemotherapy may improve outcomes, although results remain inconsistent [28–31]. The present findings suggest that reduced immunogenicity underlies the limited ICI responses in TTF-1-negative tumors, implying that additional interventions such as cytotoxic chemotherapy or radiotherapy, which can induce immunogenic cell death, may enhance the immune response in these cases.

Mollaoglu et al. reported that *NKX2-1* suppresses neutrophil recruitment in LUAD, highlighting a tumor-suppressive aspect of TTF-1 [46]. As a study directly addressing TIME features in TTF-1-negative LUAD, Tanaka et al. found that Serglycin (SRGN), secreted by TTF-1-negative lines, was associated with increased tumor PD-L1 and PD-1^+^ T-cell infiltration [47], suggesting a more immune-inflamed yet immunosuppressed TIME. Though this appears opposite to the present findings in terms of inflammation, SRGN expression did not perfectly align with TTF-1 status, and their cohort included more advanced tumors. Heterogeneity was also observed in the present cohort, with some TTF-1-negative tumors showing high TAM PD-L1/PD-L2 and vice versa. In addition, increased Tregs in TTF-1-positive tumors were suggested in both IHC and TCGA analyses, possibly due to EGFR-mutations promoting CCL22-mediated Treg recruitment [48]. These findings indicate that tumor-intrinsic mechanisms of immune evasion may also be present in TTF-1-positive tumors. Collectively, these observations suggest that both TTF-1-negative and -positive LUADs exhibit considerable intragroup immune heterogeneity.

Several limitations should be noted. First, T-cell infiltration differences had modest effect sizes, likely underpowered due to the few TTF-1-negative cases. Cell numbers alone may not fully capture immune function; functional alterations in cytokine signaling or T-cell activity could be more relevant, warranting further validation. Second, the lack of correlation between *NKX2-1* expression itself and *CD274* expression in macrophages contrasts with the IHC findings. This discrepancy likely reflects the technical limitations of single-cell data, including dropout artifacts and the difficulty in detecting subtle changes in transcription factors with low transcript abundance [49, 50]. In addition, post-translational mechanisms, such as HECW1-mediated degradation, acetylation, and phosphorylation [51–53], can contribute to the discordance between mRNA and protein levels. In contrast, the TTF-1 target gene score was more closely correlated with macrophage *CD274* expression and was consistent with the IHC results, suggesting it more accurately reflects the functional impact of TTF-1. Conversely, *PDCD1LG2* did not follow this trend, indicating it may be regulated by distinct mechanisms. Third, scRNA-seq and TCGA cohorts were stratified by median TTF-1 target gene score, although this approach has some limitations. Given the low actual prevalence of TTF-1-negative tumors, the low-score group likely includes a substantial number of TTF-1-positive tumors, limiting the specificity of this classification. Larger cohorts with matched IHC and transcriptomic datasets will be needed for clearer interpretation.

Finally, our prior work showed that granulocyte macrophage colony-stimulating factor (GM-CSF) from LUAD cells induces PD-L1 in macrophages, and this effect was blocked by GM-CSF neutralization [11]. TTF-1-positive cell lines secreted more GM-CSF than TTF-1-negative ones, consistent with the findings of Wood et al., who reported that TTF-1 induces GM-CSF expression [54]. Inaba et al. also recently reported that GM-CSF induces PD-L2 expression in dendritic cells via PU.1 [55], a transcription factor predominantly expressed in macrophages. These findings suggest that GM-CSF may contribute to the upregulation of PD-L1/PD-L2 in TAMs associated with TTF-1-positive tumors. PD-L1 in macrophages can also be induced by various other factors, including IL-10, IL-27, TNF-α, CXCL8, Toll-like receptor signaling, secreted phosphoprotein 1 (SPP1), and NF-κB activation [56], although their effects are likely context-dependent. In contrast, PD-L2 regulation remains less defined. Whereas the present data highlight IFN-γ as a key factor, multiple pathways may regulate the TTF-1-associated expression of PD-L1/PD-L2 in TAMs.

## Conclusions

TTF-1-negative LUAD exhibited lower T-cell infiltration and decreased expression of PD-L1 and PD-L2 in TAMs. These findings suggest that immune responses are attenuated in TTF-1-negative tumors, which may contribute to their poorer clinical outcomes. Further research is needed to better define the immunosuppressive features of the TIME in TTF-1-negative LUAD.

## Supplementary Information

Below is the link to the electronic supplementary material.

Supplementary file1 (DOCX 121 KB)

## Data Availability

The data supporting the findings of this study are available from the corresponding author upon reasonable request.
