# Supplementary material for: The expression of PD-1 ligands in the immune microenvironment was altered in TTF-1-negative lung adenocarcinoma
Source: Hum Cell. 2025 Aug 18;38(5):146. doi: 10.1007/s13577-025-01275-y (PMC12361300; doi:10.1007/s13577-025-01275-y)
Supplement: Supplementary file 1 — Supplementary file1 (DOCX 121 KB) [file 13577_2025_1275_MOESM1_ESM.docx]

**SUPPLEMENTARY MATERIALS**

**Title:**

Expression of PD-1 ligands in the immune microenvironment was altered in TTF-1-negative lung adenocarcinoma

**Journal:**

*Human Cell*

**Authors:**

Hiroyuki Yamada, Hiromu Yano, Eri Matsubara, Shukang Zhao, Yusuke Shinchi, Cheng Pan, Takamasa Koga, Kosuke Fujino, Yukio Fujiwara, Koei Ikeda, Yoshihiro Komohara^*^, Makoto Suzuki

^*^Corresponding author at: Department of Cell Pathology, Graduate School of Medical Sciences, Kumamoto University, 1-1-1, Honjo, Chuo-ku, Kumamoto 860-8556, Japan

E-mail address: ycomo@kumamoto-u.ac.jp (Y. Komohara)

Supplementary Table 1. Univariate and multivariate Cox regression analyses for recurrence-free survival

|  | Univariate | | | Multivariate | | |
| --- | --- | --- | --- | --- | --- | --- |
|  | HR | 95% CI | *p*-value | Adjusted HR | 95% CI | *p*-value |
| Age | | | | | | |
| ≥65 vs. <65 | 1.325 | 0.754–2.331 | 0.33 |  |  |  |
| Gender | | | | | | |
| Female vs. Male | 2.311 | 1.389–3.845 | 0.001 | 1.473 | 0.798–2.720 | 0.22 |
| Smoking | | | | | | |
| BI ≥600 vs. <600 | 2.481 | 1.522–4.045 | <0.001 | 1.825 | 0.993–3.352 | 0.053 |
| Pathological stage | | | | | | |
| II–IV vs. 0–I | 7.311 | 4.440–12.039 | <0.001 | 6.673 | 3.950–11.273 | <0.001 |
| EGFR gene status | | | | | | |
| Mutant vs. Wild type | 0.628 | 0.383–1.028 | 0.064 | 1.461 | 0.802–2.662 | 0.22 |
| TTF-1 expression | | | | | | |
| Positive vs. Negative | 0.357 | 0.200–0.637 | <0.001 | 0.615 | 0.321–1.177 | 0.14 |

Note: Underline indicates statistically significant.

Abbreviations: HR, hazard ratio; CI, confidence interval; BI, Brinkman index; EGFR, epidermal growth factor; TTF-1, thyroid transcription factor-1.

Supplementary Table 2. Logistic regression analyses for predictors of high PD-L1 MPS (≥50%)

|  | Univariate | | | Multivariate | | |
| --- | --- | --- | --- | --- | --- | --- |
|  | OR | 95% CI | *p*-value | Adjusted OR | 95% CI | *p*-value |
| Age | | | | | | |
| ≥65 vs. <65 | 1.265 | 0.700–2.291 | 0.44 |  |  |  |
| Gender | | | | | | |
| female vs. male | 0.604 | 0.351–1.035 | 0.067 | 0.732 | 0.344–1.555 | 0.41 |
| Smoking | | | | | | |
| BI ≥600 vs. <600 | 0.642 | 0.367–1.116 | 0.12 | 1.120 | 0.507–2.505 | 0.78 |
| Pathological stage | | | | | | |
| II–IV vs. 0–I | 0.541 | 0.280–1.028 | 0.063 | 0.693 | 0.348–1.372 | 0.29 |
| EGFR gene status | | | | | | |
| mutant vs. wild type | 1.935 | 1.128–3.345 | 0.017 | 1.392 | 0.745–2.605 | 0.30 |
| TTF-1 expression | | | | | | |
| positive vs. negative | 3.926 | 1.662–10.378 | 0.003 | 2.969 | 1.178–8.239 | 0.026 |

Note: Underline indicates statistically significant.

Abbreviations: PD-L1, programmed cell death-1 ligand 1; MPS, macrophage proportion score; OR, odds ratio.

Supplementary Table 3. Logistic regression analyses for predictors of high PD-L2 MPS (≥50%)

|  | Univariate | | | Multivariate | | |
| --- | --- | --- | --- | --- | --- | --- |
|  | OR | 95% CI | *p*-value | Adjusted OR | 95% CI | *p*-value |
| Age | | | | | | |
| ≥65 vs. <65 | 0.681 | 0.368–1.240 | 0.21 |  |  |  |
| Gender | | | | | | |
| female vs. male | 0.517 | 0.298–0.889 | 0.018 | 0.800 | 0.363–1.790 | 0.58 |
| Smoking | | | | | | |
| BI ≥600 vs. <600 | 0.501 | 0.285–0.874 | 0.015 | 0.906 | 0.392–2.097 | 0.82 |
| Pathological stage | | | | | | |
| II–IV vs. 0–I | 0.221 | 0.107–0.434 | <0.001 | 0.269 | 0.128–0.545 | <0.001 |
| EGFR gene status | | | | | | |
| mutant vs. wild type | 2.477 | 1.431–4.340 | 0.001 | 1.681 | 0.878–3.231 | 0.12 |
| TTF-1 expression | | | | | | |
| positive vs. negative | 3.818 | 1.652–9.631 | 0.003 | 2.179 | 0.849–5.938 | 0.11 |

Note: Underline indicates statistically significant.

Abbreviations: PD-L2, programmed cell death-1 ligand 2


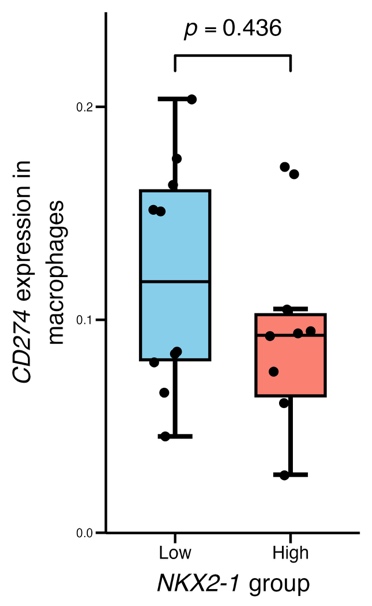

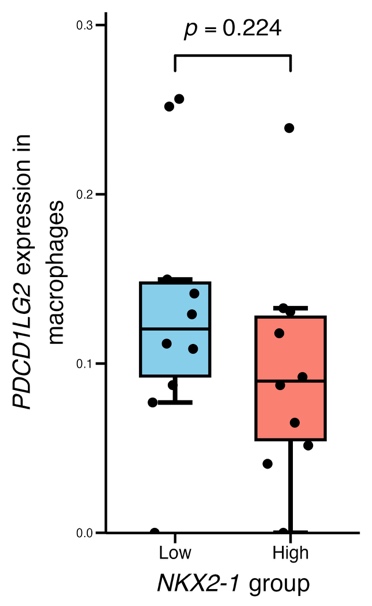


*NKX2-1*

*NKX2-1*

Supplementary Figure 1.

Comparison of *CD274* and *PDCD1LG2* expressions in macrophages between *NKX2-1*-high and -low groups. Patients were stratified by median *NKX2-1* expression in tumor cells. Comparisons were performed using the Mann-Whitney *U* test.
